# Immunotherapy in Early Parkinson's Disease: A Biomarker-Driven Trial Framework for Secondary Prevention

**DOI:** 10.7759/cureus.96090

**Published:** 2025-11-04

**Authors:** Roman Kniazev

**Affiliations:** 1 Department of Immunology, Institute for Molecular Medicine, Huntington Beach, USA

**Keywords:** biomarkers, disease modification, immunotherapy, monoclonal antibody, parkinson’s disease, prodromal pd, secondary prevention, vaccine, α-synuclein

## Abstract

Parkinson's disease (PD) is characterized by α-synuclein aggregation and progressive dopaminergic neuron loss that begins years before motor symptoms appear. This prolonged premotor phase creates an opportunity for intervention before extensive neurodegeneration. Biomarkers, including α-synuclein seed amplification assays, standardized peripheral tissue biopsies (e.g., skin biopsies for phosphorylated α-syn), neuroimaging, fluid biomarkers, genetics, and digital measures, now detect prodromal PD biology in at-risk individuals and enable secondary-prevention-style trials. Immunotherapy targeting misfolded α-synuclein is a leading disease-modification strategy. Passive approaches (monoclonal antibodies) and active vaccines have shown target engagement in preclinical models and early trials. Phase 1/2 α-synuclein antibody trials have shown mainly mild-to-moderate infusion reactions and no reproducible CNS inflammatory toxicity. Although primary efficacy endpoints have not yet been achieved overall, prasinezumab has indicated exploratory slowing in faster progressors and digital measures. MEDI1341 has demonstrated Phase 1 target engagement with acceptable safety, and Lu AF82422 has shown Phase 1 target engagement, along with promising subgroup trends in MSA, and scheduled Phase 3. Meanwhile, cinpanemab was negative. However, signals of slowed progression in subgroups underscore the importance of treating earlier and selecting patients with biomarker-confirmed pathology. This review synthesizes evidence supporting early, biomarker-anchored intervention and the status of α-synuclein immunotherapies. Treating PD at the prodromal stage aims to preserve vulnerable neurons and alter the disease course; continued advances in biomarkers and trial design are essential to realize this potential. We emphasize biomarkers that verify α-synuclein pathology during the asymptomatic phase and argue for a secondary-prevention model that treats the underlying biology before symptoms to maximize disease-modifying potential. We also propose a practical, biomarker-guided trial blueprint for secondary prevention, with explicit screening funnels and endpoint hierarchies.

## Introduction and background

Parkinson's disease (PD) involves pathological aggregation of α-synuclein and the progressive loss of dopaminergic neurons; current dopaminergic therapies are purely symptomatic [1-3]. Pathological changes in PD likely begin years before diagnosis; α-synuclein aggregates have been found in olfactory, enteric, and peripheral tissues (consistent with Braak staging), and motor signs emerge only after ~50%-60% of nigral neurons are already lost [3]. Consistent with the Putative Dual-Hit Hypothesis, misfolded α-syn pathology may originate at two peripheral interfaces, the olfactory mucosa and the enteric nervous system, and ascend via the olfactory tract and vagal/autonomic pathways to the brainstem and substantia nigra; this periphery-first model provides mechanistic support for detecting pathology years before diagnosis and motivates peripheral assays (skin p-α-syn, cerebrospinal fluid (CSF)/tissue seed amplification assays (SAA)) in secondary-prevention designs [3]. This premotor interval provides a critical window for intervention. Biologically, the goal of intervening premotor is to neutralize small, mobile α-syn oligomers and seeds that mediate prion-like cell-to-cell propagation; limiting these species should reduce templated aggregation and the subsequent formation of less-reversible fibrils and Lewy pathology.

α-Synuclein immunotherapy, delivered passively via monoclonal antibodies or actively via vaccination, has been shown to reduce pathological α-synuclein spread in animal models [4] and is supported by contemporary reviews [3]. Early-phase trials in manifest PD indicate acceptable safety and target engagement (e.g., clear changes in α-synuclein biomarkers), but no definitive clinical benefit has been observed to date [1,5].

Biomarkers can now identify PD-related pathology in at-risk or prodromal individuals, enabling prevention-style trials [6-11]. For example, α-synuclein SAA in CSF and peripheral tissues can detect misfolded α-synuclein in prodromal PD with high specificity [6]. Molecular imaging (e.g., dopamine transporter single-photon emission CT (DAT-SPECT)) and peripheral biopsies (skin nerve fibers immunostained for phosphorylated α-syn) further corroborate the early pathology [8,10,11].

Notably, the manifest-PD trials were conducted after diagnosis; emerging evidence suggests greater benefit may be achievable by treating earlier and enriching cohorts for those with active α-synuclein pathology [12]. Indeed, post hoc analyses from an anti-α-synuclein antibody trial (PASADENA) suggest that patients with faster progressing early PD experienced less motor decline on treatment compared to those receiving placebo [13]. Early active immunization programs in PD have demonstrated robust antibody responses in early/very early PD cohorts [14,15], and vaccine-induced antibodies have shown a reduction in CSF α-syn seeding activity ex vivo, indicating target engagement [16]. Small immunomodulatory studies, such as those involving sargramostim (granulocyte-macrophage colony-stimulating factor, GM-CSF), report immune shifts toward increased regulatory T cells, accompanied by exploratory motor signals [17]. Lessons from Alzheimer’s disease (AD) support intervening earlier in the pathogenic process: the antiamyloid antibody lecanemab slowed cognitive decline when given in early AD [2], whereas similar treatments failed to show benefit in late-stage disease [18].

This review outlines the case for early intervention in PD, describes biomarkers for detecting prodromal PD, and summarizes current passive and active α-synuclein immunotherapies. We focus on an asymptomatic, biomarker-verified secondary-prevention approach, treating the underlying disease biology before overt motor onset. In addition, we propose a concrete trial blueprint, including a biomarker-guided screening funnel and a hierarchy of endpoints, to facilitate the design of prevention trials in PD.

## Review

Methods

We conducted a literature search (2018-2025) on PubMed, recent neurology conference abstracts, and ClinicalTrials.gov for studies on PD biomarkers and immunotherapies. Key terms included “Parkinson's disease early intervention”, “α-synuclein immunotherapy”, and “prodromal Parkinson biomarkers”. Priority was given to recent studies on α-synuclein SAA, neuroimaging, and biofluid markers for early PD, as well as passive or active α-synuclein immunotherapies. Foundational work on PD pathology staging and neuron-loss thresholds was included for context. We also incorporated select 2024-2025 updates from high-impact sources (including recent reviews and trial readouts) to ensure currency [14]. No human or animal subjects were involved, so IRB approval was not required. When selecting sources, we prioritized peer-reviewed research from high-impact journals (those in the top quartile according to Journal Citation Reports or SCImago Journal Rank), with a few seminal older studies included for historical context. Conference abstracts and press releases are cited sparingly, primarily for very recent trial data that has not yet been fully published.

Rationale for early intervention and biomarker-driven detection

Neurodegeneration in PD is well advanced by the time of clinical diagnosis, meaning that starting a putative disease-modifying therapy at diagnosis is already an attempt to “catch up” after significant damage [11]. Prodromal PD may span a decade or more, often marked by subtle nonmotor features (e.g., hyposmia, constipation, depression, or idiopathic REM sleep behavior disorder, iRBD) [11]. Intervening before neurodegeneration crosses the threshold for motor symptoms could fundamentally alter the disease trajectory [2,13], keeping patients in a milder state for longer or even preventing clinical conversion. By the time classic motor signs appear, over half of substantia nigra dopaminergic neurons may already be lost [11], and no therapy can resurrect dead neurons. Thus, the goal is to preserve neurons by treating them earlier in the pathogenic process.

Evidence from other neurodegenerative diseases supports this strategy. In AD, biomarker-confirmed early treatment with an antiamyloid antibody (lecanemab) significantly slowed cognitive decline [2]. PD likely follows a similar principle: once motor symptoms emerge, widespread synaptic loss and exhausted compensatory mechanisms may limit observable benefit from disease-modifying therapy. Consistent with this, an exploratory analysis of the PASADENA trial of prasinezumab (an anti-α-synuclein antibody) found that patients with faster progressive early PD had less motor worsening on treatment than placebo [13], suggesting that those with active ongoing pathology derived greater benefit [1]. Taken together, these data support a secondary prevention model in PD: initiating therapy at a biomarker-verified, asymptomatic stage to slow or halt the disease before substantial clinical disability develops.

Realizing this vision depends on reliably identifying individuals with underlying PD pathology before they become symptomatic and then demonstrating that an intervention can modify biological or clinical progression. Biomarkers are the key to both. Modern assays can detect misfolded α-synuclein in patients years before PD diagnosis [6,7]. In the following, we discuss the available biomarkers for the detection of prodromal PD and how they can be used to design efficient secondary prevention trials. We then review current α-synuclein immunotherapeutic strategies that could be deployed in these biomarker-positive populations [15].

Biomarkers for presymptomatic PD detection and trial design

Secondary prevention in PD means treating the underlying disease process before clinical PD is diagnosed. In practice, this involves identifying people who have a significant risk of developing PD (due to clinical prodromal indicators or genetic factors) and have objective evidence of PD-like pathology already occurring. The most direct way to confirm prodromal PD pathology is to demonstrate misfolded α-synuclein in the nervous system via SAA or pathological tissue staining. Individuals with iRBD or marked olfactory loss, for example, have an elevated risk of PD; among such groups, those who test positive for α-synuclein aggregates (in CSF or via tissue biopsy) represent ideal candidates for an early intervention trial (see Table 1).

**Table 1:** Roles of key biomarkers in prodromal PD trials ^*^Investigational α-synuclein PET tracers; no clinically validated ligand is available as of 2025 † represents direct/primary signal; (†) represents indirect/supportive signal; - represents not applicable SAA: seed amplification assays; CSF: cerebrospinal fluid; PD: Parkinson’s disease; DAT-SPECT: dopamine transporter single-photon emission CT; α-syn PET: α-synuclein positron-emission tomography; NfL: neurofilament light; GFAP: glial fibrillary acidic protein; GBA: glucocerebrosidase; LRRK2: leucine-rich repeat kinase 2; PRS: polygenic risk score Source: [6-13]

Biomarker class	Detects α-syn biology	Detects neurodegeneration	Measures function/clinical expression	Primary trial use
SAA (CSF/tissue)	†	-	-	Inclusion gate; pharmacodynamic kinetics (e.g., time-to-threshold, Fmax)
Skin biopsy (p-α-syn)	†	-	-	Peripheral corroboration; repeatable PD readout
DAT-SPECT	-	†	-	Staging/adjudication; annualized slope as secondary progression marker
α-syn PET^*^	†	-	-	Exploratory target engagement (when validated/available)
Fluid biomarkers (NfL, GFAP, cytokines)	-	† (NfL), (†) (GFAP)	-	Safety/degeneration signal; not PD-specific; subtype context
Genetics (GBA, LRRK2, PRS)	(†)	-	-	Risk/subtype enrichment and stratification
Digital measures (wearables/phone)	-	-	†	High-frequency real-world functional endpoints

α-Synuclein SAA

SAA techniques (e.g., real-time quaking-induced conversion) can ultrasensitively detect misfolded α-synuclein by amplifying its aggregation in vitro. In prodromal cohorts, such as iRBD patients, CSF SAA is positive in most individuals and strongly predicts eventual phenoconversion to PD or a related synucleinopathy [6]. For example, in one study of iRBD, more than 90% of patients had positive CSF α-syn SAA, while almost none of the controls did [6]. This makes CSF SAA an invaluable tool for enriching trials with actual prodromal PD cases [6,12]. Blood-based SAA (plasma or saliva) is being explored as a less invasive screening method [7]; recent work has shown that misfolded α-syn seeds in blood can persist for four to seven years before PD diagnosis in some individuals [7]. However, blood SAA protocols are still being standardized [12], and CSF (or tissue) SAA remains the gold standard for confirming prodromal PD pathology [6,12]. In the context of a trial, baseline SAA kinetic parameters (e.g., the lag time of the aggregation curve or maximum fluorescence) can serve as quantitative pharmacodynamic markers to track how an immunotherapy may be altering the underlying pathology [12]. Interlaboratory variability remains nontrivial (preanalytical handling, reaction parameters, positivity thresholds); multisite trials should use locked protocols, common reagents, and blinded proficiency testing.

Peripheral Tissue Pathology

Specific peripheral tissues show PD-like α-synuclein pathology even in the prodromal stage. Pathology is defined as phosphorylated α-syn (Ser129) in autonomic nerve fibers (not total α-syn); the yield is site-dependent, with autonomically innervated regions (e.g., C7 paravertebral neck, distal thigh, distal leg) prioritized for central reading. Multicenter studies have demonstrated a high prevalence of phosphorylated α-syn in skin nerves of patients with manifest PD or iRBD, with excellent specificity versus controls [8]. In one study, >85% of iRBD patients (who all eventually developed PD or dementia with Lewy bodies) had positive skin biopsies, while healthy controls did not [8]. Such findings indicate that skin biopsy can provide pathological confirmation of prodromal PD, complementing CSF SAA. The biopsy procedure is minimally invasive and can be repeated, making it useful for both screening (to enrich a trial with actual α-synuclein pathology) and for tracking response (a reduction in skin p-α-syn burden over time could signal target engagement by the therapy). Given that iRBD carries an estimated 70% risk of conversion to a synucleinopathy over 10-15 years [11], a positive skin biopsy in an iRBD patient identifies an ultrahigh-risk subgroup that might benefit most from early treatment [8,11]. We recommend standardized biopsy handling, including obtaining two to three skin samples from consistent sites, using rigorous immunohistochemistry protocols with appropriate controls, and centralized, blinded reading, to ensure reliable results across sites.

Neuroimaging Biomarkers

While no imaging modality yet visualizes α-synuclein aggregates in living people (α-syn positron-emission tomography (PET) tracers are still experimental), other imaging modalities can detect early PD-related changes. The most widely used is dopamine transporter single-photon emission CT (DAT-SPECT), which can reveal reduced striatal dopaminergic innervation years before clinical PD. Prodromal individuals (e.g., those with iRBD or leucine-rich repeat kinase 2 (LRRK2) mutations) often have abnormal DAT scans in advance of symptoms [9]. However, DAT imaging measures neurodegeneration rather than α-syn pathology per se, and it may remain normal in very early stages. Its primary utility in asymptomatic trials is for baseline staging (to confirm whether presynaptic damage is already present) and as a secondary endpoint (annualized change in DAT signal as an objective marker of neurodegeneration) [10]. DAT-SPECT changes slowly, so it is not sufficiently sensitive to serve as a primary endpoint in short-term trials; however, it provides supportive evidence of disease progression or stabilization [10]. For example, in the PARS study, individuals with hyposmia and subtle DAT deficits had a higher rate of PD conversion over a four-year period [9]. In the current trial design, we suggest using DAT-SPECT selectively: obtain a DAT scan at baseline to stratify or characterize participants (and to exclude unexpected cases of normal tracer uptake or atypical features) and consider follow-up DAT imaging yearly as a secondary outcome to gauge neurodegenerative progression [10]. Other imaging techniques, such as cardiac metaiodobenzylguanidine scintigraphy (for autonomic denervation), advanced MRI (e.g., neuromelanin or diffusion imaging of the substantia nigra), or transcranial ultrasound, have been explored; however, their roles remain investigational. In the future, a synuclein PET ligand could revolutionize early PD trials by directly measuring cortical and subcortical α-synuclein deposition; ongoing early-phase studies of candidate tracers are eagerly anticipated.

Fluid Biomarkers (Beyond SAA)

Apart from misfolded α-syn, other biofluid markers have been studied in prodromal PD, but none have adequate standalone sensitivity or specificity. Total α-synuclein levels in CSF tend to be lower in individuals with established PD compared to controls; however, there is overlap, and no clear prodromal signal is evident. Assays for oligomeric or phosphorylated α-syn show promise but are not yet standardized [16]. Neuronal-enriched exosomal α-syn and plasma p-α-syn are emerging non-CSF candidates for scalable screening, but assays and clinical performance are not yet standardized. Neurofilament light chain (NfL), a nonspecific marker of neurodegeneration, is typically elevated in early PD and prodromal states. This elevation is informative, as it suggests an atypical Parkinsonian disorder rather than PD [11]. For instance, patients with pure autonomic failure or iRBD (prodromal synucleinopathies) have low NfL. In contrast, those with multiple system atrophy (MSA; an α-syn plus disorder) have high NfL due to more widespread cell death. Thus, NfL can be used in screening to exclude cases that are likely to have atypical pathology. Other fluid-based research (proteomics, metabolomics, autoantibody profiles) is ongoing to find predictive biomarkers, but none are ready for trial enrichment. A prudent approach is to combine a misfolded protein marker (e.g., SAA positivity indicating disease presence) with one or more downstream markers (reflecting the degree or subtype of disease activity). For example, one might require SAA confirmation to enter a trial but also measure baseline NfL or inflammation markers to characterize the cohort and potentially stratify analyses.

Genetic Markers

A subset of individuals at high risk for PD can be identified via genetics. LRRK2 and GBA mutations are the most common PD-related genetic variants; carriers often exhibit prodromal features (hyposmia, REM sleep behavior disorder) and some have detectable α-synuclein pathology before symptoms [5]. Asymptomatic carriers of high-penetrance mutations (e.g., SNCA multiplications, LRRK2 G2019S, specific GBA variants) are candidates for early intervention trials, especially if they show biomarker evidence of pathology (for example, an LRRK2 carrier who is SAA-positive). Genetics can also guide therapy matching; for instance, patients with GBA mutations may accumulate α-syn faster due to impaired lysosomal clearance, making them especially suitable for α-synuclein-targeted immunotherapy trials. Additionally, an individual's polygenic risk score for PD could be integrated with prodromal markers to refine risk prediction. In short, genetics can enrich trial populations and help define subgroups who might respond differently to specific disease-modifying strategies.

Digital Biomarkers

Subtle motor and nonmotor symptoms of PD often precede clinical diagnosis and can be captured by wearable sensors or smartphone apps. Continuous monitoring of movement, sleep, voice, and cognition in the real world yields high-frequency data that may detect microprogression, which is invisible to periodic clinical exams. For example, a smartwatch-based motor score or typing kinematics might show progressive slowing or reduced amplitude even in the prodromal stage. Digital outcomes have already proven sensitive in early PD trials. In the PASADENA trial, a sensor-based motor composite showed less worsening in the antibody-treated group than in the placebo group, despite the standard clinical score (Movement Disorder Society‐Sponsored Revision of the Unified Parkinson's Disease Rating Scale, MDS-UPDRS) not showing a significant difference [13]. In a prevention trial, a composite digital endpoint integrating metrics such as finger-tapping speed, gait rhythm, and sleep disturbance could serve as a primary outcome to indicate whether an intervention is slowing the emergence of motor features. These measures can be collected continuously or in frequent, short bursts at home, thereby improving the signal-to-noise ratio. It is crucial to ensure data quality (e.g., requiring a minimum wear time and using algorithms to detect non-wear periods or poor data) and to predefine how the digital data will be summarized (e.g., a weekly average of daily step count or a z-score combining multiple tasks) [13].

Enrichment and Trial Design Strategy

**Figure 1:**
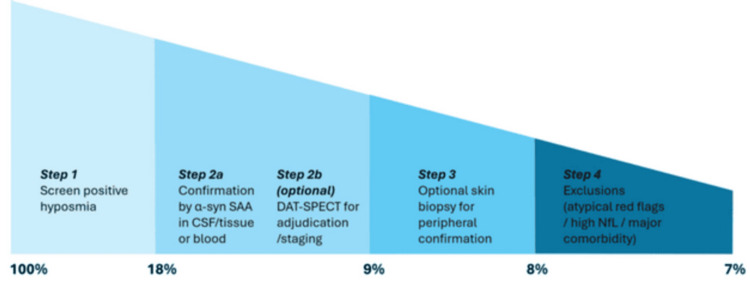
Suggested PD biomarker funnel from risk screen to enrollment Biomarker-anchored trial framework for asymptomatic/very early PD. Funnel steps reflect a periphery-first (Dual-Hit) propagation model linking early enteric/olfactory pathology to central spread. A staged funnel enriches clinically high-risk, biomarker-positive candidates for secondary prevention. Step 1: Low-cost remote risk screening (e.g., smell test, polysomnography-confirmed iRBD); expect ~15%-20% of hyposmia screen-positive results (use site-specific cutoffs). Step 2: definitive confirmation with SAA: prefer CSF RT-QuIC; tissue SAA acceptable in specialized labs. In hyposmia-selected cohorts, assume ~50% SAA-positive (base case); in iRBD, CSF SAA ≈85%-90% [6]; SAA is the primary inclusion gate [1,5]. Hyposmia-led Step 1 to Step 2 throughput is ≈7%-10%. Step 2b (optional): DAT-SPECT to adjudicate discordance and stage nigrostriatal integrity; not an eligibility gate unless explicitly used for enrichment. Adding a DAT deficit increases near-term risk (PARS-style designs) [9]. Step 3 (optional): standardized skin biopsy (cervical/distal leg) to corroborate peripheral α-syn; among SAA-positive entrants, ~90% confirmation with p-Ser129 protocols and central reads [8]. Exclude atypical features (elevated NfL, disproportionate cerebellar/autonomic signs) and significant comorbidities (~10% attrition). Net yield examples: hyposmia-led ≈7% of the original screen (0.18 × 0.50 × 0.90 × 0.90 ≈ 0.073); iRBD-led ≈70% (0.88 × 0.90 × 0.90 ≈ 0.71) [2,5]. Baselines captured for longitudinal change: SAA kinetics, digital motor/sleep metrics, and, if obtained, DAT-SPECT PD: Parkinson’s disease; iRBD: idiopathic REM sleep behavior disorder; SAA: seed amplification assays; CSF: cerebrospinal fluid; RT-QuIC: real-time quaking-induced conversion; DAT-SPECT: dopamine transporter single-photon emission CT; PARS: Parkinson-associated risk syndrome Image credit: This is an original image created by the author, synthesized from [6-11]

Our enrichment strategy begins with clinical risk assessment and culminates in the confirmation of α-syn pathology. By funneling participants toward misfolded α-syn positivity, we select those at the most significant near-term risk of conversion and most likely to benefit from α-syn-targeted therapy. This approach also standardizes baseline measures for longitudinal analyses, including CSF SAA kinetics, optional skin biopsy scores, DAT-SPECT binding (if performed), fluid markers such as NfL (typically normal in prodromal PD), and digital motor/sleep metrics (see Figure 1).

Endpoints and trial design considerations

Because phenoconversion to clinical PD is slow, diagnosis is impractical as a primary outcome within typical trial timelines [11]. We therefore propose a dual-endpoint strategy: 1) a biologically anchored composite surrogate for early decision-making and 2) long-term clinical follow-up for definitive outcomes.

Primary Endpoint (12-24 Months)

A prespecified composite biomarker outcome (either weighted or hierarchical) comprises the following: 1) change in CSF α-synuclein seeding kinetics (e.g., longer lag time or lower maximum fluorescence, indicating slower templating/propagation), 2) change in peripheral p-α-syn pathology on standardized follow-up skin biopsies, and 3) change in a validated digital motor/sleep composite capturing subtle functional decline [8,12,13]. This composite increases sensitivity to disease modification by detecting early biological and functional changes before overt symptoms [13]; improvement or stabilization over 12-24 months would indicate a treatment impact on the disease cascade.

Secondary endpoints include the individual components of the composite (each analyzed separately), the annualized change in DAT-SPECT specific binding ratio (as an objective measure of neurodegeneration rate) [10], safety/tolerability outcomes, and patient-reported outcomes (quality of life and prodromal scales). Although DAT-SPECT changes are modest over short intervals, a slower decline over approximately two years in the treated group would support a neuroprotective effect.

Long-Term Outcomes

All participants would enter an extension or observational follow-up phase to assess time-to-phenoconversion to clinical PD, adjudicated by expert criteria. Because conversion events are infrequent early on, this analysis is event-driven and may require at least five years to reach sufficient numbers [11]. This ensures that a short-term biomarker benefit translates into a meaningful clinical delay. This two-pronged design balances feasibility and rigor: short-term, biology-anchored evidence can support go/no-go decisions (and potential accelerated approval if the biomarker is reasonably likely to predict clinical benefit). At the same time, long-term follow-up confirms that biomarker improvements correlate with delayed clinical PD.

Statistical Design

A hypothetical two-arm, randomized, placebo-controlled trial in biomarker-positive prodromal PD could be powered with an 18-month composite as the primary endpoint. Illustratively, detecting a 30% slowing in this composite with 90% power at a two-sided α = 0.05 may require 80-100 participants per arm, depending on the outcome variance and correlations among components. Long-term follow-up would be event-driven: in an iRBD-enriched cohort converting at ~5%-10% per year, ~150 phenoconversion events would provide robust power to detect a hazard ratio of ~0.70. Efficiency can be increased through adaptive features, including interim futility/efficacy analyses, blinded sample-size reestimation, and enrichment strategies that preferentially recruit faster progressors informed by observational data. Missing data and intercurrent events would be addressed with prespecified estimands, for example, mixed models for repeated measures on continuous composites and appropriate censoring in time-to-event models when symptomatic PD therapy is initiated. These figures are illustrative; final parameters would be refined with pilot data and simulations.

Trial Endpoints and Analysis

We suggest moving away from “time-to-clinical conversion” as the sole primary endpoint and instead centering on biology that is measurable in one to two years. The primary endpoint would be a prespecified composite (as described above) that captures multiple dimensions of disease activity (protein aggregation, neuronal damage, and subtle clinical function). We predefine a composite primary endpoint (CPE) that combines three domains: 1) CSF SAA kinetics (longer lag time and lower maximum fluorescence denote improvement), 2) skin p-α-syn burden (lower standardized score denotes improvement), and 3) a validated digital motor/sleep composite (lower worsening denotes improvement). Each component will be standardized to a z-score with direction harmonized, and combined as a weighted sum $\text{CPE} = w_1 \cdot z_{\text{SAA}} + w_2 \cdot z_{\text{Skin}} + w_3 \cdot z_{\text{Digital}}$ with prespecified base-case weights w1 = 0.50, w2 = 0.25, w3 = 0.25, reflecting biological proximity to the putative mechanism and expected measurement precision. The covariance matrix of component changes will be estimated to account for intercomponent correlation. Inference on the CPE utilizes a mixed model for repeated measures with an unstructured covariance and prespecified covariates. Sensitivity analyses will include 1) equal-weight CPE, 2) a rank-based O’Brien-type composite, 3) a win-ratio analysis, and 4) a hierarchical gatekeeping test (SAA → skin → digital) to ensure robustness if components are highly correlated. If pairwise correlation exceeds 0.7, we will report component-wise results and the hierarchical analysis alongside the CPE. A fixed weighting or hierarchical testing schema can be used to combine these measures while controlling multiple comparisons. The key requirement is directional coherence: the components should all trend in a hopeful direction if an actual disease-modifying effect is present. This biomarker-first design enables earlier, data-driven decisions on whether a therapy is impacting the disease process. Key secondary endpoints include safety, patient-reported outcomes, and the evaluation of each component of the composite individually. All participants will be followed long-term, with time-to-clinical PD conversion as a crucial supportive endpoint to confirm that any biomarker gains translate into a tangible clinical benefit over time (see Table 2).

**Table 2:** Trial biomarker matrix for an asymptomatic/very-early PD secondary-prevention trial This table outlines the role and timing of key biomarkers from screening through on-treatment follow-up. α-syn PET is investigational. DAT-SPECT is optional and primarily for staging/adjudication purposes α-Syn SAA: α-synuclein seed amplification assays; CSF: cerebrospinal fluid; PD: Parkinson’s disease; IHC: immunohistochemistry; DAT-SPECT: dopamine transporter single-photon emission CT; PET: positron-emission tomography; GFAP: glial fibrillary acidic protein; GBA: glucocerebrosidase; LRRK2: leucine-rich repeat kinase 2; PRS: polygenic risk score; LP: lumbar puncture Source: [6,8-10,12,13]

Biomarker/modality	Sample/tool	What it measures	Best use in trials	Strengths	Limitations
α-Syn SAA (CSF)	Lumbar CSF	Templated α-syn misfolding (pathology presence)	Inclusion/enrichment, pharmacodynamics (kinetics)	High specificity; detects prodromal biology	LP required; lab harmonization needed
α-Syn SAA (tissue)	Skin/other tissue lysate	Templated misfolding in peripheral tissue	Inclusion confirmation; PD readout	Peripheral access; repeatable	Fewer labs; standardization evolving
Skin biopsy (p-α-syn IHC)	Skin punch	Aggregated/phosphorylated α-syn in autonomic fibers	Inclusion; serial peripheral target engagement	Minimally invasive; topographic mapping	Sensitivity varies by lab/site
DAT-SPECT	Imaging	Presynaptic dopaminergic terminal integrity	Baseline staging; progression secondary endpoint	Widely available; validated	Not α-syn specific; modest responsiveness
Exploratory α-syn PET	Imaging	Putative α-syn aggregates	Regional focus; exploratory PD readout	Direct biology (if validated)	Early-phase; tracer variability
NfL (plasma/CSF)	Blood/CSF	Axonal injury	Safety/degeneration signal; subtype differentiation	Easy sampling; prognostic context	Not PD-specific; often normal early
Inflammatory markers (e.g., GFAP, cytokines)	Blood/CSF	Glial activation/inflammation	Stratify immunomodulatory responders; combo readout	Mechanistic insight	Heterogeneous; low specificity
Genetics (GBA, LRRK2, PRS)	Blood/saliva	Risk/biology subtype	Enrichment, stratification, precision combos	Anchors precision medicine	Risk ≠ destiny; counseling needed
Digital motor/speech/sleep	Wearables/phone	High-frequency function (real world)	Sensitive progression endpoint	Scalable; patient-centric	Standardization; data quality

Decision Rules

Prespecify biomarker-triggered actions to ensure consistent responses and maintain safety focus. For example, if a participant's CSF SAA result turns persistently negative during the trial, investigators could reassess that individual's status while continuing therapy per protocol (since a negative conversion might indicate a profound treatment effect but also warrants verification). If a skin biopsy sample has a low nerve fiber density that limits interpretability, a repeat biopsy can be performed at an alternate standardized site. An excessive decline in DAT-SPECT binding in a treated participant could trigger a safety review (e.g., to assess for inflammation or an amyloid-related imaging abnormality-like phenomenon, as seen with antibody therapies in other diseases). A rising NfL level might prompt evaluation for atypical Parkinsonism or an intercurrent illness causing neuroaxonal injury. If wearable device data show nonadherence (insufficient data capture), sites should retrain and reengage the participant to improve compliance. Together, these rules operate on how biomarkers guide enrollment, monitor pharmacodynamic effects, and ensure safety, leveraging their dual roles (eligibility inclusion and on-treatment readouts) to increase sensitivity to actual disease-modifying effects.

Clinical-stage passive immunotherapy targeting α-synuclein (monoclonal antibodies)

Passive immunotherapy involves administering laboratory-made antibodies that bind to misfolded or pathological forms of α-synuclein, aiming to neutralize toxic aggregates, block their cell-to-cell spread, and promote clearance via microglia or peripheral mechanisms. Monoclonal antibodies are intended to bind extracellular oligomeric/protofibrillar conformers, which are thought to be the most transmissible and toxic species, thereby curbing interneuronal seeding and promoting clearance. They offer precise dosing (typically given as periodic infusions) and are immediately active (no need to wait for the body to generate antibodies). Below, we summarize the key passive immunotherapies tested in PD, focusing on their performance in trials and the lessons learned so far.

Prasinezumab (PRX002/RG7935)

Prasinezumab is a humanized IgG1 monoclonal antibody directed against the C-terminus of α-synuclein. In α-syn transgenic mouse models, C-terminal-targeted antibodies reduced the spread of α-syn aggregates and preserved motor function [3,4]. In the Phase 2 PASADENA trial, prasinezumab was tested in early-stage, drug-naïve PD patients. The primary endpoint, a 12-month change in the MDS-UPDRS total score, was not met, with no significant difference from placebo in overall motor progression [1,13]. However, several secondary and exploratory outcomes favored prasinezumab: treated patients showed smaller worsening on the MDS-UPDRS Part III (motor exam), trends toward less decline in striatal DAT-SPECT binding, and better outcomes on selected digital motor measures [5]. A post hoc subgroup of “fast progressors” showed ~35% less motor worsening over 12-15 months with prasinezumab compared to placebo [1]. These findings suggest no broad efficacy in unselected early PD but potential benefit in biologically active or rapidly progressing subgroups. The Phase 2b PADOVA trial in early PD patients already on symptomatic therapy reportedly also missed its primary endpoint, though detailed efficacy data are pending. Prasinezumab has been generally well-tolerated, with mainly mild to moderate infusion reactions and no major CNS inflammatory events reported. Target engagement is confirmed by dose-dependent increases in plasma total α-synuclein (consistent with antibody-antigen complex formation) and a CSF-to-serum ratio of ~0.3%, typical for an IgG1 antibody [1]. Prasinezumab appears safe and engages its target, with suggestive signals of slowed progression in certain subgroups. Future trials should enrich participants with confirmed α-synuclein pathology (e.g., SAA-positive prodromal individuals) and employ sensitive biomarkers and digital endpoints to maximize the likelihood of detecting disease-modifying effects [1,3,5].

Cinpanemab (BIIB054)

This antibody was designed to target extracellular aggregated α-syn (binding to the N-terminus residues ~1-10) in the hope of slowing disease progression. Phase 1 data were encouraging (acceptable safety, expected pharmacokinetics). However, in the Phase 2 SPARK trial (early PD over 18 months), cinpanemab did not significantly slow MDS-UPDRS progression, nor did it affect DAT imaging or other measures; the trial was stopped early for futility. In terms of safety, the antibody was generally well tolerated, with no significant safety concerns reported. The following question has been raised regarding interpretation: why might it have failed? Several factors are possible: 1) epitope targeting: the N-terminus of α-syn can be partially hidden in specific aggregate conformations, whereas the C-terminus (targeted by prasinezumab) is often exposed and modified in pathological fibrils. An N-terminal antibody might miss the dominant toxic species in human PD; 2) timing and biology: even “early” PD in SPARK had already exhibited substantial neurodegeneration; with limited antibody brain penetration, achieving a meaningful effect might have been challenging once the pathology was well entrenched; and 3) cohort heterogeneity and endpoints: PD progression varies, and if only a subset of patients would respond, a group-level effect could be diluted in a relatively short trial with coarse clinical endpoints. The lesson is not that immunotherapy cannot work; it is that we need to apply it to the right patients and at the right time. Future trials should move into the prodromal phase (biomarker-confirmed individuals before extensive neurodegeneration), verify target engagement with pharmacodynamic readouts (e.g., changes in SAA kinetics or skin p-α-syn levels), and choose epitopes that target exposed, pathogenic species of α-syn. Pairing traditional clinical scales with sensitive digital and biomarker outcomes will help ensure that real biological effects are detected even if clinical changes are subtle.

MEDI1341 (Terepercimab)

MEDI1341 is a high-affinity monoclonal antibody developed by AstraZeneca (Cambridge, United Kingdom) in partnership with Takeda (Tokyo, Japan). It selectively targets aggregated α-synuclein and was engineered for two key features: improved brain penetration and reduced effector function, the latter designed to minimize microglial activation and associated neuroinflammation. In a Phase 1 trial (2019-2020) involving both healthy volunteers and patients with PD, MEDI1341 demonstrated an acceptable safety profile, with no drug-related serious adverse events reported. Notably, there was clear evidence of target engagement: treatment produced dose-dependent reductions in “free” CSF α-synuclein levels and achieved a CSF: serum ratio of approximately 0.1%-0.2% [5]. Despite these promising pharmacological signals, as of 2025, no Phase 2 efficacy study has been publicly initiated, suggesting the program is either on hold or awaiting further strategic decisions. MEDI1341 established proof-of-principle that an engineered antibody can bind central α-synuclein in humans while minimizing inflammation. Next-generation antibodies should follow this model, optimizing brain delivery and tolerability. Tolerability must also incorporate biomarker-enriched, earlier stage cohorts and utilize pharmacodynamic readouts (e.g., SAA kinetics or skin biopsy measures) to link target engagement with actual disease modification. A Phase 2 study of MEDI1341, also known as TAK 341, in multiple system atrophy did not achieve its primary or secondary endpoints, leading Takeda to announce it will not pursue further development for MSA. Efficacy results for Parkinson's disease have not been reported.

Lu AF82422

Lu AF82422 is an IgG1 monoclonal antibody developed by Lundbeck (Denmark) that binds both monomeric and aggregated α-synuclein, though its precise epitope has not been disclosed. In a Phase 1 single-ascending-dose trial involving healthy volunteers and patients with PD or MSA, the antibody showed no safety concerns, linear pharmacokinetics, and CSF exposure within the expected low percentage of serum levels [5]. The Phase 2 study in MSA did not meet the primary endpoint but showed encouraging subgroup trends; Lundbeck plans to proceed to Phase 3. MSA, a rapidly progressive synucleinopathy characterized by oligodendroglial α-syn inclusions, provides an opportunity to detect treatment effects over shorter timelines than in PD. For PD, future development may logically focus on biomarker-enriched subgroups, such as SAA-positive prodromal subjects or defined genetic carriers, and could explore combinations with complementary approaches (e.g., immunomodulators).

Summary and Perspective

Lu AF82422 appears tolerable and achieves CNS exposure; however, its disease-modifying potential remains unproven until the MSA trial is completed and PD-specific studies are pursued. MSA Phase 2 has been concluded, showing a missed primary endpoint but supportive subgroup trends. A Phase 3 program is proceeding, yet no efficacy results for Parkinson's disease have been reported. Across passive immunotherapy programs, the pharmacologic prerequisites have been met, antibodies can be dosed chronically, penetrate the CNS at measurable levels, and alter α-synuclein profiles in CSF or plasma. The remaining challenge is to translate target engagement into clear neuroprotection. The mixed results so far reinforce the rationale for testing these agents earlier, at the biomarker-confirmed prodromal stage, where salvageable neuronal substrate exists and effect sizes may be larger. Future trials should also incorporate more sensitive outcomes, including SAA kinetics, skin p-α-syn burden, and digital composites, given that conventional clinical scales often miss subtle but meaningful early changes [13].

Clinical-stage active immunotherapies targeting α-synuclein (vaccines)

Active immunotherapy aims to stimulate the patient's own immune system to produce antibodies against α-synuclein. Potential advantages of a vaccine approach include the production of long-lasting antibodies (after an initial series of injections, only occasional boosters may be needed) and the ability to generate a polyclonal antibody response that targets multiple epitopes of α-synuclein. The challenge is to break immune tolerance to a native protein without inducing autoimmunity. Below, we review key active immunotherapy programs in PD.

AFFITOPE Vaccines (PD01A and PD03A)

These are short synthetic peptides (AFFITOPE® technology, Affiris AG, Vienna, Austria) conjugated to a carrier (keyhole limpet hemocyanin) and adjuvanted with aluminum, designed to mimic α-synuclein epitopes while minimizing T-cell autoreactivity. The goal is to induce a robust antibody response against extracellular pathogenic α-syn. In Phase 1 trials in early PD, both vaccines were well tolerated, with mainly injection-site reactions or mild flu-like symptoms reported [5]. Immunogenicity was robust, as most participants developed antibodies that bind to aggregated α-syn. PD01A targets a C-terminal epitope of α-syn; PD03A targets an N-terminal epitope preferentially expressed on oligomeric α-syn. An open-label extension suggested that treated patients had relatively stable motor scores over approximately one year and possible reductions in CSF oligomeric α-syn levels. However, these studies were small and uncontrolled [5]. Active vaccination in PD thus appears feasible and safe. AC Immune (Lausanne, Switzerland) has since reformulated PD01A as ACI-7104 with a liposomal delivery platform to enhance immunogenicity. A Phase 2 trial (named “VacSYn”) is underway, enrolling prodromal and very early PD participants with documented α-syn pathology [14]. Interim 2024 data showed no safety issues and a nearly 20-fold increase in anti-α-syn antibody titers in vaccine recipients [14,15]. If the blinded results demonstrate even a trend toward slower clinical or biomarker decline, it will warrant larger trials and bolster enthusiasm for vaccines in PD.

UB-312 (Vaxxinity, Dallas, Texas​​​​)

UB-312 is another peptide-based vaccine designed to elicit antibodies against pathological α-syn species. In a Phase 1 trial, UB-312 was well tolerated and generated high, sustained antibody titers that preferentially bind to aggregated α-syn. Importantly, antibodies from vaccinated patients were shown to blunt α-syn seeding activity in patient CSF samples, demonstrating target engagement in vivo [16]. This is a critical proof-of-mechanism, indicating that vaccine-induced antibodies can reach the CNS and neutralize toxic α-syn seeds. For a secondary prevention setting, maintaining sufficiently high antibody titers is crucial (since only a small fraction of circulating IgG enters the brain). The Phase 1 data suggest UB-312 can achieve those titers. Ongoing studies will need to assess whether these immune responses translate into slower disease progression. The safety profile must remain excellent given the intent to treat asymptomatic individuals; thus far, no serious safety signals have emerged. If UB-312 can safely sustain strong, specific antibody levels, and if those levels correlate with improved biomarker outcomes (e.g., slowed SAA aggregation or reduced skin pathology), a gentler clinical trajectory could become a viable tool for secondary prevention in PD.

ACI-7104 (AFFITOPE Next-Generation)

ACI-7104 is the liposome-formulated version of the AFFITOPE PD01A vaccine. As mentioned, the ongoing Phase 2 trial (VacSYn) is one of the first to explicitly target a prodromal/very early PD population, including patients with iRBD or early PD who have biomarker evidence of α-syn pathology [14]. Interim results reported in 2025 indicate that the vaccine induces a strong immune response, with mean anti-α-syn antibody titers more than 20-fold higher than those in the placebo group after four immunizations [15]. Full efficacy readouts (for motor ratings, DAT imaging, and biomarkers such as SAA) are pending. A robust and sustained antibody response is a prerequisite for disease modification; the early data are encouraging in this regard. Even a modest slowing of progression in this Phase 2 would likely catalyze larger trials. Looking ahead, AC Immune is also developing an α-syn DNA vaccine, which could potentially offer an even more durable immune response with fewer doses, if the peptide approach proves successful and safe.

Other approaches include emerging active immunization strategies, such as viral-vector-mediated vaccines (e.g., AAV vectors encoding α-syn mimetic antigens to elicit an immune response) and novel antibody formats, such as nanobodies (single-domain antibodies), which may penetrate tissues more effectively and access intracellular α-syn aggregates. A complementary approach is immune modulation, altering the immune environment to favor clearance and neuroprotection rather than targeting α-syn directly. For example, a small trial of sargramostim (GM-CSF) in PD demonstrated an increase in regulatory T cells and suggested a mild improvement in motor function in treated patients [17]. The premise is that reducing neuroinflammation or enhancing peripheral immune regulation could slow neurodegeneration or amplify the effects of protein-targeted therapies. In the future, combination regimens may emerge, such as pairing an α-syn-targeted vaccine or antibody with an immunomodulator to dampen harmful inflammation or promote repair.

Active immunization against α-synuclein is feasible and, to date, generally safe across multiple platforms. Several vaccine programs induce high titers of antibodies specific for pathogenic α-syn species. Notably, UB-312 has demonstrated direct target engagement in patients, reducing CSF seeding activity [16], providing crucial proof of mechanism. The remaining question is efficacy: can these vaccines actually slow the progression of the disease? This will require longer, biomarker-rich trials. If successful, vaccination could become a cornerstone of secondary prevention: at-risk individuals might one day receive an α-syn vaccine in midlife, mount a lasting antibody response that suppresses α-syn aggregation, and thereby prevent the development of motor symptoms associated with PD. That paradigm shift, from treating PD after it arises to preventing it in those with early pathology, is the ultimate goal of immunotherapy in prodromal PD.

Discussion

Immunotherapy in early or presymptomatic PD rests on a strong biological rationale, but several hurdles remain before we see apparent clinical success. First, the biomarker-clinical disconnect: therapies can have robust biological effects (e.g., reducing CSF α-syn seeding activity) without immediate discernible changes on coarse clinical scales over one to two years. Early-stage PD progresses slowly and heterogeneously, so traditional measures, such as the UPDRS, may miss subtle changes [13]. Trials should therefore pair periodic clinical exams with sensitive, mechanism-specific endpoints, such as serial CSF SAA for protein aggregation, neuroimaging for neuronal integrity, and continuous digital monitoring of real-world motor function, so that biological benefits are not lost in the noise of clinical variability [13].

Disease Heterogeneity

PD is a syndrome with multiple subtypes and pathogenic pathways; α-syn aggregation might not be the dominant driver in every patient. In unselected cohorts, this dilutes any treatment signal. The solution is precision enrollment: require biomarkers that confirm α-syn pathology (e.g., a positive CSF or tissue SAA) to ensure the target is present [6,12], analogous to requiring amyloid PET positivity in Alzheimer's trials [1,3]. Further stratification by genetic or molecular subtypes (for instance, testing a combination of immunotherapy and an LRRK2 inhibitor in LRRK2 mutation carriers) can help identify subgroups most likely to respond.

Mechanistic Uncertainties

Most antibodies primarily act on extracellular α-syn, whereas most pathogenic α-syn is intracellular. A benefit may still arise if extracellular binding shifts the equilibrium and promotes the clearance of intracellular species, but it is not guaranteed. Truly addressing intracellular aggregates may require alternative approaches (e.g., intrabodies or small molecules). Additionally, the “peripheral sink” hypothesis, which states that antibodies could sequester α-syn in the bloodstream and pull equilibrium out of the brain, remains unproven. Improving CNS delivery of antibodies (through engineering or intrathecal administration) might increase efficacy, but must be balanced against safety and practicality. In summary, success will likely hinge on treating the right biology at the right time and using next-generation agents tailored to the pathogenic species and locations of α-syn, all while measuring outcomes with the precision needed to detect changes in early disease.

Neuroinflammation and Combination Strategies

PD pathology is accompanied by a host of immune changes: microglial activation, cytokine release, and adaptive immune infiltration. Clearing misfolded α-syn should, in theory, remove a key driver of this inflammation. However, if antibody therapy engages Fc receptors on microglia, it might actually increase inflammatory responses; this is why some antibodies (e.g., MEDI1341) are engineered with reduced effector function. A rational next step is to combine an α-synuclein-targeted approach with an immunomodulatory therapy that tilts the balance toward neuroprotection. Early signals with sargramostim (GM-CSF), which increases regulatory T-cells, showed a hint of motor benefit and reduced inflammation markers [17], supporting the idea that modulating the immune state can positively influence PD progression. In short, addressing the cause (protein aggregation) and the consequence (inflammation) together may yield additive or synergistic neuroprotection.

Next-Generation Trial Design

The field is beginning to shift trials into prodromal and very early PD, with enrichment by biomarker status. Ongoing studies are enrolling patients with markers such as SAA positivity or genetic risk who have not yet developed significant symptoms, and are using novel outcomes (including digital measures) to capture changes. Trial designs are also becoming more adaptive and stratified, for example, planning subgroup analyses by genotype or adding combination arms. A conceivable blueprint is to randomize SAA-positive iRBD subjects to immunotherapy versus placebo, use a sensitive composite endpoint (digital plus biomarker) at 18-24 months for the primary analysis, and include an add-on agent (such as an LRRK2 inhibitor) in a subset of participants with relevant mutations. In parallel, other therapeutic modalities (such as antisense oligonucleotides to reduce α-syn production or bispecific antibodies that engage microglia for clearance) are being explored; these could be combined with vaccines or antibodies to attack aggregation from multiple angles.

Endpoints for Prodromal Trials

Conventional clinical outcomes (e.g., time to initiation of levodopa or change in the UPDRS) are not well-suited for asymptomatic cohorts. Trials should employ milestone events (like diagnosis conversion) only as long-term endpoints and use validated surrogate markers in the interim. Regulatory precedent exists for this approach. In AD, trials in early stages have been evaluated based on biomarker changes (amyloid PET reduction, plasma p-tau improvements), with the understanding that these predict clinical benefit [2]. In PD, extensive observational studies (such as the Parkinson's Progression Markers Initiative) are helping establish which biomarkers predict progression, thereby strengthening the argument that a therapy-driven change in those markers will translate into clinical benefit. As the evidence linking biomarker outcomes to clinical outcomes solidifies, regulators may become more amenable to approving therapies based on a composite biomarker endpoint in high-risk individuals, with post-approval clinical verification.

Ethics and Practical Constraints

Moving intervention upstream to at-risk individuals raises critical ethical considerations. Disclosing a “PD-pathology-positive” status (for example, telling someone with iRBD that they have a biomarker indicating underlying PD biology) can cause anxiety, affect insurability, and carry stigma. Trial protocols must include thorough counseling and transparent consent processes that explain the implications of biomarker results. Often, studies choose not to explicitly disclose specific results (e.g., informing an individual only that they did or did not qualify, rather than saying “your SAA was negative”) to avoid undue distress or false reassurance. Another challenge is the long-time horizons: a prevention trial may need to follow participants for many years to confirm clinical benefit. This necessitates well-coordinated multicenter networks, as well as creative trial designs (e.g., adaptive and enrollment of high-progression subsets) to maintain momentum and feasibility.

Momentum, Not Verdicts

The initial wave of immunotherapy trials (e.g., cinpanemab, prasinezumab) taught us that the concept is biologically plausible but execution sensitive. A negative or neutral trial outcome should not be seen as a refutation of the strategy, but rather as guidance to refine our approach [3]. The field is indeed pivoting: moving toward biomarker-enriched and earlier stage cohorts, incorporating digital endpoints, and learning from each attempt. For instance, trials in prodromal LRRK2 carriers and an anti-α-syn antibody trial in iRBD are underway, reflecting the hypothesis that treating individuals with confirmed pathology before diagnosis is the most effective approach.

Beyond Single-Agent Thinking

It is increasingly recognized that PD is unlikely to be cured by a single mechanism intervention. Pathological protein aggregation, neuroinflammation, mitochondrial dysfunction, and other processes all contribute to neuron loss. Thus, ultimate therapy may require a combination approach, such as an α-synuclein immunotherapy to reduce toxic aggregates, along with a neurotrophic or anti-inflammatory agent to support neuronal health. Combination trials are more complex, but preclinical models suggest that targeting multiple nodes of the disease cascade may offer additive benefits. Over time, we may see trials testing immunotherapy backbones with various adjuncts to address the multifactorial nature of PD.

Design Trials for Prevention, Not Rescue

Waiting for patients to convert clinically makes sense from a statistical power standpoint, but from a biological perspective, it means playing catch-up. If we can “see the fire” of α-syn pathology smoldering years before diagnosis (via SAA or biopsy), we should attempt to extinguish it early. This calls for trial endpoints that track the fire's intensity rather than waiting for the house to burn down. A surrogate-first endpoint, composed of complementary biomarkers, can collectively inform us within one to two years whether the disease process is slowing. This endpoint includes CSF seeding activity to reflect molecular templating, skin p-α-syn to reflect peripheral spread, DAT or other imaging to reflect neuron loss, and a robust digital measure for clinical function. No single marker is definitive on its own, but concordance across domains would provide a compelling signal amidst the noise. Enriching the trial population (so that nearly everyone enrolled truly has underlying synuclein pathology and is on the verge of conversion) dramatically increases the ability to detect a signal. Adaptive trial features can further shorten the timeline by dropping ineffective treatments early and concentrating on promising signals. We would still track traditional clinical outcomes in the longer term because, ultimately, preventing symptoms is the goal. However, we would not “hold progress hostage” to the slow march of clinical onset. In essence, we should design prevention trials that provide actionable answers on a molecular level within a couple of years, while still validating the clinical benefits over the subsequent years.

What Success Will Require? 

We must treat the right people: those with biomarker-confirmed α-syn pathology and minimal neurodegeneration, and at the right time, before motor onset in the prodromal phase. We must also measure the proper outcomes, using sensitive biological and functional markers rather than infrequent clinic-based scores. Aligning the population, timing, and endpoints in this way gives immunotherapy the best chance to demonstrate disease modification and translate biomarker improvements, such as slower SAA seeding, reduced skin p-α-syn burden, and stabilized digital motor/sleep composites, into tangible patient benefits. If these elements are in place, we can realistically hope to see a disease-slowing effect that changes the course of PD for the first time [1].

## Conclusions

PD begins years before symptoms, leaving a clear window to prevent or slow damage. Biomarkers can identify the right individuals and measure change. These include CSF, tissue, or blood α-synuclein SAA to confirm pathology; standardized skin biopsy for phosphorylated α-syn; and, when helpful, DAT-SPECT and validated digital motor or sleep measures. α-Synuclein immunotherapies (antibodies and vaccines) are generally safe and show target engagement, but starting after diagnosis has yielded limited clinical benefit. The most promising strategy is to treat biomarker-confirmed, minimally symptomatic, or even asymptomatic individuals and to read out biology early using SAA kinetics, skin biopsy scores, and a robust digital composite; imaging can support staging. Next steps include developing better brain penetrant medicines, standardizing and sharing assays, and conducting prevention-oriented, adaptive trials that enroll high-risk, pathology-positive participants. Success requires coupling the biomarker funnel with a prespecified composite endpoint, combining SAA kinetics, skin p-α-syn, and a validated digital composite to deliver actionable readouts within 12-24 months, while phenoconversion is tracked long term.
